# Commentary: Serum total bilirubin with hospital survival in adults during extracorporeal membrane oxygenation

**DOI:** 10.3389/fmed.2022.1022207

**Published:** 2022-09-28

**Authors:** Chunxia Wang, Yucai Zhang

**Affiliations:** ^1^Department of Critical Care Medicine, Shanghai Children's Hospital, Shanghai Jiao Tong University School of Medicine, Shanghai, China; ^2^Pediatric Extracorporeal Life Support Center, Institute of Pediatric Infection, Immunity, and Critical Care Medicine, Shanghai Jiao Tong University School of Medicine, Shanghai, China; ^3^Institute of Pediatric Critical Care, Shanghai Jiao Tong University, Shanghai, China

**Keywords:** bilirubin, hospital survival, ECMO, venoarterial, venovenous, timing

## Introduction

Extracorporeal membrane oxygenation (ECMO) is increasingly being used as a life-saving therapy for patients with cardio-pulmonary dysfunction who have failed conventional treatment (1). ECMO support removes carbon dioxide from the patient's blood and returns oxygenated blood to the patient (2, 3). However, ECMO-associated complications—e.g., activation of complement and contact systems leading to cytokine release—pose significant risks to successful patient management. Moreover, complications such as acute kidney injury, pneumonia, sepsis, and bleeding are common during ECMO support (4). Recently, ECMO-associated liver injury or direct hyperbilirubinemia (DHB) has been receiving more attention in both adult and pediatric populations due to its association with high mortality (5–9). It is noteworthy that the incidence of DHB (defined as >3 mg/dL of serum bilirubin) was as much as 73% in patients undergoing venoarterial ECMO (VA-ECMO) (7). Monitoring of serum total bilirubin (TBIL) could therefore be useful for assessing prognosis in patients receiving ECMO.

## Subsections relevant for the subject

Huang et al. (9) reported on the important role of serum TBIL in predicting survival in adults on ECMO support. Their work produced several key findings: First, the patients who died within 28 days had twice the peak level of TBIL as those who survived. Multivariate logistic regression analysis indicated that a high peak TBIL was correlated with mortality risk at 28 days and total mortality after adjusting for age, ECMO mode, sepsis, lactate level, and APACHE II score (a general measure of disease severity). Second, hyperbilirubinemia after ECMO initiation was a risk factor for worse prognosis. Third, TBIL, but not aspartate transaminase (AST) or alanine aminotransferase (ALT), was an important indicator of ECMO-associated liver injury, and therefore a significant predictor of prognosis, with the cutoff value of 65 μmol/L. Fourth, peak TBIL was associated with coagulation dysfunction, and was correlated with prothrombin time (PT), activated partial thromboplastin time (APTT), prothrombin time activity (PTA), and fibrinogen (FIB) levels. Fifth, there was a potential association between the sequential organ failure assessment (SOFA) score and hyperbilirubinemia after ECMO initiation. Taken together, these results demonstrate that TBIL is an optimal biomarker for assessing hospital survival in adults during ECMO.

## Discussion

An increased bilirubin concentration during the first 48 h after admission was significantly associated with adverse long-term outcome in patients with moderate to severe acute respiratory distress syndrome (ARDS) (10). Patients with severe ARDS and increased bilirubin had high mortality rates at 24, 48, and 72 h after venovenous ECMO (VV-ECMO) initiation. However, increased bilirubin values before ECMO initiation was not associated with increased in-ICU mortality (6). ECMO-associated DHB was also associated with high mortality in children receiving ECMO support (8), as reported by Huang et al. (9). Round-the-clock monitoring of serum bilirubin would therefore be beneficial for the precision management of patients receiving ECMO.

Bilirubin is formed in hepatic Kupffer cells, in the monocytic macrophages of the spleen, and in bone marrow. In a normal adult, ~250–300 mg of bilirubin is formed every 24 h, at a production rate of 3.8 mg/kg (11). DHB during VA-ECMO could be caused by the lack of pulsatile flow to end organs or by medication-induced liver injury. Moreover, circuit-induced hemolysis or a massive pathological intravascular hemolysis can contribute to the development of high mixed DHB in VA-ECMO patients. Besides DHB, ECMO-induced hemolysis leads to the most severe form of pump head thrombosis (12), and the rate of hemolysis is decreased with centrifugal pumps compared to roller pumps (13). More importantly, the causes of DHB in patients under ECMO support should be analyzed case-by-case.

Compared with non-survivors, bilirubin levels in survivors trended down on the day of ECMO initiation (14), suggesting that the timely and effective removal of bilirubin is necessary for improving outcomes. The hemolysis index (HI) assay on Abbott's Alinity CI system allows accurate determination of plasma free hemoglobin concentrations, enabling the assessment of ECMO-associated hemolysis and thereby helping to prevent and manage hemolysis-induced DHB (15, 16). Advanced strategies for the management of liver failure, including artificial liver support systems, plasma exchange, and bilirubin adsorption, are gradually seeing increased usage in critically ill patients. Huang et al. (9) showed that the SOFA score was potentially associated with hyperbilirubinemia occurrence after ECMO initiation, with a prediction accuracy of 0.800.

Currently, predicting the occurrence of ECMO-associated DHB still poses a challenge for intensivists. Integrating artificial liver support systems into the ECMO circuit might be useful for improving hospital survival of patients under ECMO support, but few data are available and these issues need further study.

## Author contributions

CW and YZ drafted and edited the manuscript. Both authors contributed to the article and approved the submitted version.

## Funding

This work was supported by the Science and Technology Commission of Shanghai Municipality (20Y11901300) and National Natural Science Foundation of China (82171729). CW was supported by Shanghai Municipal Education Commission-Gaofeng Clinical Medicine Grant No. 20171928.

## Conflict of interest

The authors declare that the research was conducted in the absence of any commercial or financial relationships that could be construed as a potential conflict of interest.

## Publisher's note

All claims expressed in this article are solely those of the authors and do not necessarily represent those of their affiliated organizations, or those of the publisher, the editors and the reviewers. Any product that may be evaluated in this article, or claim that may be made by its manufacturer, is not guaranteed or endorsed by the publisher.
